# Impact of anticoagulants on the clinical outcomes of colonic diverticular bleeding comparing warfarin and direct oral anticoagulants

**DOI:** 10.1038/s41598-022-21166-8

**Published:** 2022-10-07

**Authors:** Li-sa Chang, Tsutomu Nishida, Kana Hosokawa, Yoshifumi Fujii, Naoto Osugi, Aya Sugimoto, Kaori Mukai, Dai Nakamatsu, Kengo Matsumoto, Shiro Hayashi, Masashi Yamamoto, Masami Inada

**Affiliations:** Department of Gastroenterology, Toyonaka Municipal Hospital, 4-14-1 Shibahara, Toyonaka, Osaka 560–8565 Japan

**Keywords:** Gastroenterology, Risk factors

## Abstract

Recently, direct oral anticoagulants (DOACs) have been widely used as antithrombotic agents to replace warfarin, but their clinical impact in patients with gastrointestinal bleeding is unclear. We compared the effects of warfarin and DOACs on the outcomes of patients with colonic diverticular bleeding. The patients were divided into warfarin and DOAC groups. We compared the clinical outcomes and the effect of the DOAC dosing and examined any readmissions due to colonic diverticular bleeding within 1 year. A total of 95 events (warfarin group: *n* = 43 and DOAC group: *n* = 52) were included. Compared with the warfarin group, the DOAC group was significantly older, had a lower rate of concomitant antiplatelet agents, and a shorter hospital stay, but no significant differences were found in the other clinical outcomes. Thirty-seven patients (71.2%) in the DOAC group had appropriate dosing, whereas 15 patients (28.9%) had an inappropriate dose. The patients with overdose or contraindications had significantly lower minimum hemoglobin levels. In the univariate analysis, prior hospitalization for colonic diverticular bleeding was a significant predictor of readmission. Compared with warfarin, patients with colonic diverticular bleeding treated with DOACs were older and had shorter hospital stays, and the inappropriate use of DOACs may worsen outcomes.

## Introduction

Since the 1950s, warfarin, a traditional vitamin K antagonist, has been widely used to prevent thromboembolism; however, its several drawbacks have prompted the development of more convenient drugs^1^. With the introduction of the first direct oral anticoagulant (DOAC) dabigatran in 2010, several researchers have emphasized several advantages of DOACs over warfarin, including rapid onset of action, absence of the effect of vitamin K, fewer drug interactions, and predictable pharmacokinetics^2^. However, DOACs can lead to several problems such as contraindication in patients with mechanical heart valves, valvular atrial fibrillation (AF) and severe renal dysfunction, and higher costs when compared with warfarin^3^. Recent studies have shown the superiority or noninferiority of DOACs to warfarin for their antithrombotic effect and reduced bleeding risk^4–6^ in patients with AF. On the other hand, these pivotal studies have also suggested a significant increase in gastrointestinal (GI) bleeding in patients treated with DOACs. Brodie et al. reported that GI bleeding in patients taking DOACs may be less severe when compared with those taking warfarin^7^. However, the safety of DOACs with respect to bleeding risk remains controversial. Therefore, in clinical practice, physicians often adjust the dose of DOACs that are not in compliance with either the approved label or clinical guidelines, which take into consideration various factors, such as age, body weight, or renal function. Several population-based studies have indicated that the off-label use of DOACs is associated with an increase in clinically significant bleeding^8^, and their impact on GI bleeding has been of great interest to gastroenterologists. In the context of these circumstances, this study originally aimed to assess the bleeding profile of DOAC therapy compared to that of warfarin therapy. GI bleeding refers to various forms and sources of bleeding in the GI tract, and its heterogeneity in the patient population often makes it difficult to eliminate confounding factors that may affect the results. Since colonic diverticular bleeding accounts for 30% of cases of lower GI bleeding^9^ and is estimated to account for a large portion of annual healthcare costs^10,11^, we decided to focus on the difference in colonic diverticular bleeding, a representation of lower GI bleeding, between warfarin and DOACs. In the present study, we examined the effect of DOACs and warfarin on the clinical outcomes of colonic diverticular bleeding. In addition, we assessed the impact of the inappropriate use of DOACs on the clinical outcomes of colonic diverticular bleeding.

## Materials and methods

This was a single-center, retrospective study. We surveyed consecutively hospitalized patients with a diagnosis of colonic diverticular bleeding among those who presented with lower GI bleeding at Toyonaka Municipal Hospital from November 2010 to November 2021. The patients were selected from the database, and the data were collected from the electronic medical records of our hospital (MegaOak online imaging system, NEC, Japan). The patients were evaluated and diagnosed using computed tomography (CT), colonoscopy, and laboratory data. Colonic diverticular bleeding was diagnosed based on the criteria described by Jensen et al.^12^, including (1) active bleeding of colonic diverticulosis observed by colonoscopy, (2) a nonbleeding vessel or an adherent clot in the diverticula observed by colonoscopy, (3) the absence of blood in the terminal ileum and no other major gastrointestinal lesions observed on colonoscopy, (4) colonic diverticulosis with extravasation as observed by enhanced CT, and (5) blood collection in the colon noted on plain CT with evidence of bleeding and without abdominal pain but no evidence of bleeding from other major GI lesions in cases for which an emergent colonoscopy was difficult. The indication for hospitalization of a patient with colonic diverticular bleeding was determined at the discretion of the attending physician based on the disease severity and the patient’s background. In the present study and among these patients, we enrolled those who were on oral anticoagulant therapy at the time of admission.

### Outcomes

We compared the clinical course of events between the DOAC and warfarin groups based on the administration of oral anticoagulants on admission. The primary outcomes included the duration of hospital stay and fasting, blood transfusion and units of red blood cells, hemoglobin levels on admission and the minimum hemoglobin levels during the hospital stay, rebleeding events during hospitalization, and readmission due to recurrence (30 days and 1 year). The secondary outcomes were the clinical outcomes in DOAC-treated patients who had different doses and with different risk factors for readmission within 1 year.

### Dosages and therapeutic control of anticoagulant drugs

The warfarin-treated group was categorized into three groups according to their status and depending on the prothrombin time-international normalized ratio (PT- INR) control: within, below, or above the therapeutic range. The therapeutic range refers to PT-INR values ranging from 2.0 to 3.0 (1.6–2.6 in patients over 70 years), as defined in the 2020 Japanese Circulation Society Guideline on Pharmacotherapy of Cardiac Arrhythmias^13^. All four types of DOACs have specific dose reduction criteria based on the patient’s renal function, age, and body weight, and as summarized in the guidelines mentioned above, which describe five subgroups: appropriate standard-dose, appropriate low-dose, overdose, underdose, and contraindication. In the present study, the appropriate-dose group was defined as those patients who were taking the recommended dose and that met the reduction criteria, including appropriate standard-dose and appropriate low-dose. The underdose group included those patients who were taking a reduced dose of DOACs, determined based on the judgment of the attending physician and on the patient's background, although it was recommended that these patients take a standard dose. The overdose group included those patients who were taking a standard dose, although it was recommended that they take a reduced dose.

### Ethical considerations

This study was conducted in accordance with the Declaration of Helsinki, and approval was obtained from the Institutional Review Board of Toyonaka Municipal Hospital (No. 2022-03-06). The requirement for informed consent was waived via the *opt-out* method on our hospital website.

### Statistical analysis

Medians and interquartile ranges (IQRs) are reported for continuous variables. Categorical variables are summarized as frequencies (percentages). Fisher’s exact tests were used to evaluate the differences in the categorical variables, and the differences in the categorical variables were evaluated for statistical significance by the Wilcoxon signed-rank sum test. Univariate logistic analysis logistic regressions were performed to determine risk factors for readmission in colonic diverticular bleeding. All calculated P values were two-sided, and a *P* value < 0.05 was considered statistically significant. All statistical analyses were performed using JMP statistical software (ver. 15.2.1, SAS Institute Inc., Cary, NC, USA).

## Results

**Figure 1:**
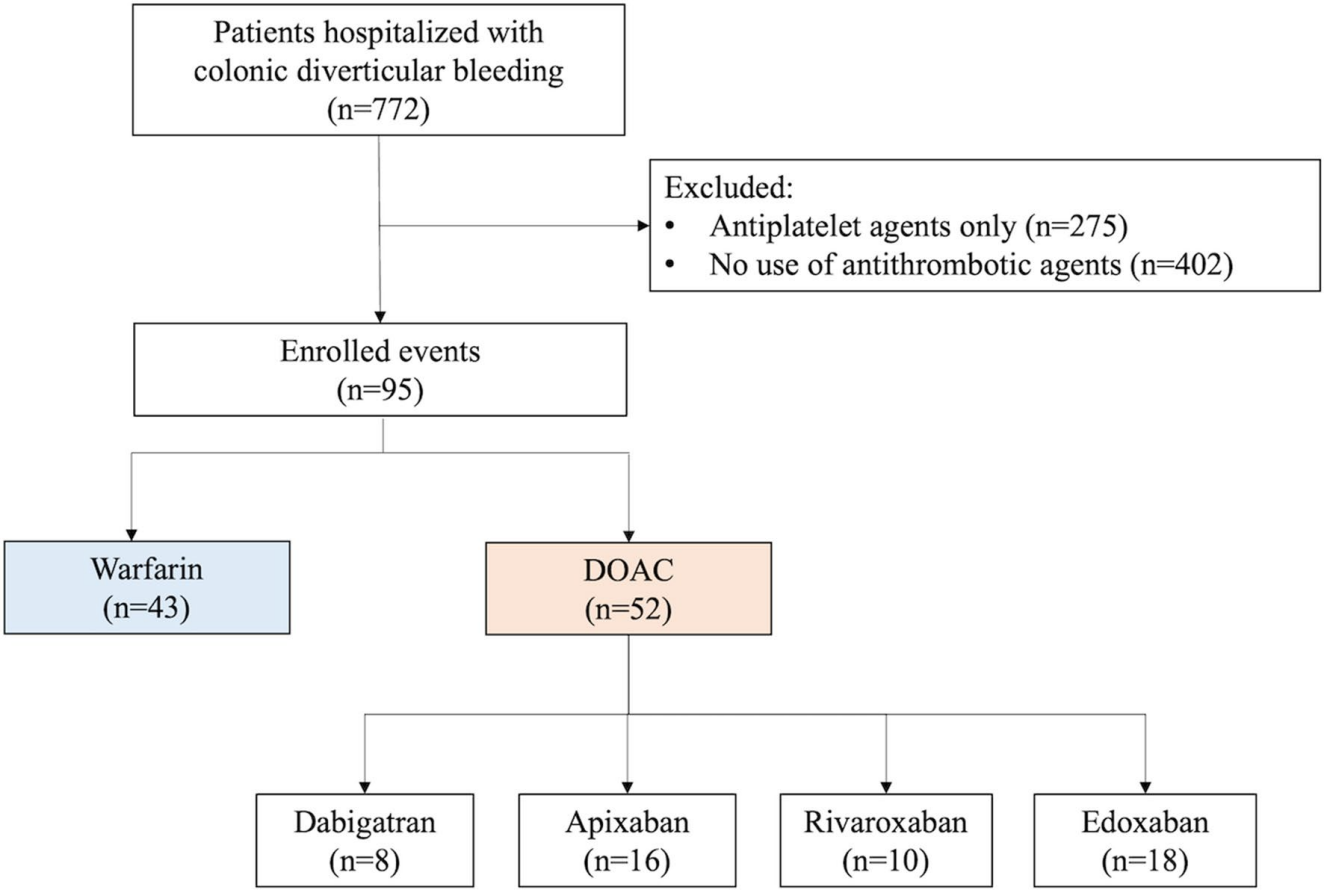
Flowchart of patient selection.

The flowchart of the patient selection is shown in Fig. 1. A total of 772 patients with a diagnosis of colonic diverticular bleeding were admitted to our hospital from November 2010 to November 2021. We excluded 275 patients on antiplatelet therapy but not on anticoagulant therapy and 402 patients who did not take any antithrombotic agents. Finally, we enrolled and analyzed a total of 95 events in patients who were exposed to oral anticoagulant therapy at the time of admission, including 49 recurrent cases in the same patients during the study period. Among them, 52 patients (54.7%) were taking DOACs (dabigatran: *n* = 8, apixaban: *n* = 16, rivaroxaban: *n* = 10, or edoxaban: *n* = 18), and 43 patients (45.3%) were taking warfarin (Fig. 1).

The clinical characteristics of the DOAC group vs. warfarin group are summarized in Table 1. The DOAC group was significantly older (84 years vs. 79 years), and patients who were over 80 years were significantly more dominant in the DOAC group (67.3% vs. 34.9%) than in the warfarin group. The warfarin group had significantly higher PT-INR values (2.21 vs. 1.17). The use of concomitant antiplatelet agents was significantly higher in the warfarin group than in the DOAC group (34.9% vs. 7.7%). The doses of anticoagulants are shown in Table 2. In the DOAC group, 37 patients (71.2%) received a per-label dose, including appropriate standard-dose and low-dose, and the other 15 patients (28.9%) received an off-label dose, including underdose, overdose, and contraindication. In the warfarin group, 60.5% (*n* = 26) were within, 14.0% (*n* = 6) were below, and 25.6% (*n* = 11) were above their therapeutic PT-INR range.

**Table 1:** Baseline characteristics of the patients on anticoagulant therapy.

	Warfarin group	DOAC group	*P* value
Event number	43	52	
Recurrent cases during the study period	22	27	1.000
2 events	7	9	1.000
3 and more events	15	18	1.000
Male sex, *n* (%)	22 (51.2)	32 (61.5)	0.4055
Age, median year (IQR)	79 (74, 81)	84 (78, 87)	0.0037
Age ≥ 80 years, *n* (%)	15 (34.9)	35 (67.3)	0.0020
BMI (kg/m^2^), median (IQR)	23.0 (19.5, 25.9)	23.8 (20.6, 26.0)	0.7649
eGFR (mL/min/1.73 m^2^), median (IQR)	49.5 (37.4, 64.1)	52.3 (35.1, 65.4)	0.6403
CCr (mL/min), median (IQR)	46.6 (32.0, 59.0)	52.0 (33.2, 62.9)	0.4024
PT-INR, median (IQR)	2.21 (1.83, 2.64)	1.17 (1.08, 1.27)	< .0001
HAS-BLED score*, median (IQR)	3 (2, 4)	3 (2, 4)	0.2736
**Concomitant medications**			
Antiplatelet agents, *n* (%)	15 (34.9)	4 (7.7)	0.0015
NSAIDs, *n* (%)	11 (25.3)	13 (25.0)	1.0000

*A scoring system was developed to assess the 1 year risk of major bleeding in patients taking anticoagulants for atrial fibrillation.

DOAC: direct oral anticoagulant, BMI: body mass index, eGFR: estimated glomerular filtration rate, CCr: creatinine clearance, PT-INR: prothrombin time-international normalized ratio, NSAIDs: nonsteroidal anti-inflammatory drugs.

**Table 2:** Dosage and therapeutic ranges of DOACs and warfarin.

**Warfarin group**	*n* = 43
Within the therapeutic range, *n* (%)	26 (60.5)
Below the therapeutic range, *n* (%)	6 (14.0)
Above the therapeutic range, *n* (%)	11 (25.6)
**DOAC group**	*n* = 52
Per-label use (appropriate), *n* (%)	37 (71.2)
Standard-dose, *n* (%)	14 (26.9)
Low-dose, *n* (%)	23 (44.2)
Off-label use (inappropriate), *n* (%)	15 (28.9)
Underdose, *n* (%)	10 (19.2)
Overdose, *n* (%)	3 (5.8)
Contraindication, *n* (%)	2 (3.8)

The primary outcomes of the present study are shown in Table 3. The period of hospitalization was significantly longer in the warfarin group than in the DOAC group (12 days vs. 9 days). The percent of patients whose withdrawal periods of anticoagulants were longer than 5 days was also higher in the warfarin group (76.2% vs. 25.5%), but there were no thrombogenic events in either group.

**Table 3:** Comparison of the clinical outcomes of patients taking warfarin vs. DOAC.

	Warfarin group	DOAC group	*P* value
Event number	43	52	
Hemoglobin levels on admission, median (IQR)	10.8 (9.5, 13.3)	11.1 (9.2, 12.8)	0.6699
Minimum hemoglobin levels, median (IQR)	8.8 (7.6, 10.8)	9.1 (7.2, 11.2)	0.8781
Blood transfusion, yes, *n* (%)	19 (44.2)	17 (32.7)	0.2917
Units of blood transfusion, median (IQR)	0 (0, 4)	0 (0, 3.5)	0.1808
Period of fasting (days), median (IQR)	4 (2, 5)	3 (2, 4)	0.2715
Period of hospitalization (days), median (IQR)	12 (9, 16)	9 (8, 11.8)	0.0063
Withdrawal of anticoagulants ≥ 5 days, *n* (%)	32 (76.2)	12 (25.5)	< 0.0001
Rebleeding during hospitalization, *n* (%)	5 (11.6)	6 (11.5)	1.0000
Readmission due to diverticular bleeding, *n* (%)	9 (20.9)	14 (27.0)	1.0000
30-day readmission, *n* (%)	1 (2.3)	3 (5.8)	0.6239
1-year readmission, *n* (%)	12 (27.9)	14 (28.0)	1.0000
Early colonoscopy*^1^ on admission, n (%)	11 (25.6)	10 (19.2)	0.4695
Elective colonoscopy*^2^, *n* (%)	17 (39.5)	18 (34.6)	0.6725
Interventional radiology, *n* (%)	1 (2.3)	1 (1.9)	1.0000

*^1^A colonoscopy was performed within 24 h of the initial visit.

*^2^A colonoscopy was performed more than 24 h after the initial visit but prior to discharge.

Next, because of the significant difference in age between the two groups, we divided the patients into two groups by age: below 80 years and over 80 years. The comparison of the clinical outcomes of the different age groups is summarized in Table 4. Of note, there were no significant differences in the period of hospitalization and withdrawal of anticoagulants in either group based on age. In the warfarin group, the patients who were over 80 years of age had worse hemoglobin levels on admission (9.7 g/dL vs. 12.2 g/dL) and minimum hemoglobin levels during hospitalization (7.8 g/dL vs. 10.1 g/dL). The warfarin-treated patients over 80 years old received more blood transfusions and required more units of blood transfusions than the patients below 80 years of age (80% vs. 25%, 4 units vs. 0 units). The patients in the DOAC group over 80 years of age showed significantly lower hemoglobin levels on admission than those below 80 years of age (10.2 g/dL vs. 11.5 g/dL); however, the older patients had similar clinical outcomes to the younger patients with respect to blood transfusion. Unlike the warfarin group, the DOAC group had significantly higher overall and 1-year readmission rates (37.1% vs. 5.9%). Similarly, in an age-specific analysis, the warfarin group had a significantly higher transfusion rate and transfusion units than the DOAC group for those aged 80 years and older. However, there were no differences in the transfusion rates and transfusion units for those younger than 80 years, although they had higher rates of concomitant antiplatelet agent use than the DOAC group. In addition, regardless of age, the warfarin group had a higher rate of anticoagulant withdrawal for more than 5 days.

**Table 4:** Comparison of the patients younger than and older than 80 years of age.

	Warfarin group			DOAC group		
	Below 80 years (*n* = 28)	Over 80 years (*n* = 15)	*P* value	Below 80 years (*n* = 17)	Over 80 years (*n* = 35)	*P* value
Hemoglobin levels on admission, median (IQR)	12.2 (10.7, 14.2)	9.7 (8.5, 9.9)	0.0002	11.5 (9.9, 14.5)	10.2 (8.7, 11.9)	0.0230
Minimum hemoglobin levels, median (IQR)	10.1 (8.6, 12.0)	7.8 (6.9, 8.2)	0.0011	9.6 (8.4, 12.1)	8.7 (7.2, 10.5)	0.2046
Blood transfusion, yes, *n* (%)	7 (25.0)	12 (80.0)*	0.0010	4 (23.5)	13 (37.1)*	0.3667
Units of blood transfusion, median (IQR)	0 (0, 3.5)	4 (2, 4)†	0.0131	0 (0, 2)	0 (0, 4)†	0.5183
Concomitant antiplatelet agents, *n* (%)	11 (39.3)	4 (26.7)	0.5118	0 (0)	4 (11.4)	0.2901
Period of fasting (days), median (IQR)	4.5 (2, 5)	3 (2, 4)	0.6127	4 (3, 5)	3 (2, 4)	0.1113
Period of hospitalization (days), median (IQR)	12 (9, 15.8)	11 (9, 19.0)	0.8580	10 (8, 11.5)	9 (7, 12.0)	0.5360
Withdrawal of anticoagulants ≥ 5 days, *n* (%)	21 (77.8)§	11 (73.3)‡	1.0000	5 (29.4)§	7 (23.3)‡	0.7334
Rebleeding during hospitalization, *n* (%)	3 (10.7)	2 (13.3)	1.0000	4 (23.5)	2 (5.7)	0.0808
Readmission due to diverticular bleeding, *n* (%)	8 (28.6)	4 (26.7)	1.0000	1 (5.9)	13 (37.1)	0.0206
30-day readmission, *n* (%)	0 (0)	1 (6.7)	0.3488	0 (0)	3 (8.6)	0.5423
1-year readmission, *n* (%)	8 (28.6)	4 (26.7)	1.0000	1 (5.9)	13 (37.1)	0.0206
Early colonoscopy on admission, *n* (%)	9 (32.1)	2 (13.3)	0.2765	6 (35.3)	4 (11.4)	0.0616
Elective colonoscopy, *n* (%)	9 (32.1)	8 (53.3)	0.2060	9 (52.9)	9 (25.7)	0.0680
Interventional radiology, *n* (%)	1 (3.6)	0 (0.0)	1.0000	1 (5.9)	0 (0.0)	0.3269

Comparison of warfarin and DOACs among patients aged older than 80 years, *P = 0.0121, †*P* = 0.0110, ‡*P* = 0.0029.

Comparison of warfarin and DOACs among patients aged younger than 80 years, §*P* = 0.0038.

Table 5 shows the clinical outcomes in the warfarin group with different PT-INR ranges: PT-INR < 3 or PT-INR ≥ 3. There were no significant differences in the clinical outcomes between the two groups. Next, a subgroup analysis was conducted to assess the results with different DOAC doses. The results of the DOAC group with different doses are shown in Table 6. The clinical outcomes of the off-label DOAC group were comparable with those of the per-label group. As shown in Table 6, the combined population of the overdose and contraindication subgroups had significantly lower minimum hemoglobin levels and longer hospitalization times than the per-label group.

**Table 5:** Clinical outcomes in warfarin-treated patients with different PT-INR values.

	PT-INR < 3 (*n* = 35)	PT-INR ≥ 3 (*n* = 8)	*P* value
Hemoglobin levels on admission, median (IQR)	11.5 (9.5, 13.5)	9.9 (8.8, 10.6)	0.0888
Minimum hemoglobin levels, median (IQR)	9.2 (7.8, 10.8)	8.0 (6.9, 8.7)	0.1112
Blood transfusion, yes, *n* (%)	14 (40)	5 (63)	0.4319
Units of blood transfusion, median (IQR)	0 (0, 4)	3 (0.5, 4)	0.1946
Concomitant antiplatelet agents, *n* (%)	11 (31.4)	4 (50.0)	0.4188
Period of fasting (days), median (IQR)	4 (2, 5)	3.5 (2, 4)	0.2395
Period of hospitalization (days), median (IQR)	12 (10, 18)	8.5 (7.3, 14.8)	0.0824
Withdrawal of anticoagulants ≥ 5 days, *n* (%)	25 (73.5)	7 (87.5)	0.6545
Rebleeding during hospitalization, *n* (%)	5 (14.3)	0 (0.0)	0.5648
Readmission due to diverticular bleeding, *n* (%)	11 (31.4)	1 (12.5)	0.4071
30-day readmission, *n* (%)	1 (2.9)	0 (0.0)	1.0000
1-year readmission, *n* (%)	11 (31.4)	1 (12.5)	0.4071
Early colonoscopy on admission, *n* (%)	10 (28.6)	1 (12.5)	0.6563
Elective colonoscopy, *n* (%)	16 (45.7)	1 (12.5)	0.1193
Interventional radiology, *n* (%)	1 (2.9)	0 (0.0)	1.0000

**Table 6:** Clinical outcomes in patients receiving DOACs at different doses.

	Per-label*^1^ (*n* = 37)	Off-label*^2^ (*n* = 15)	*P* value*	Overdose + Contraindicatio*n* (*n* = 5)	*P* value**
Hemoglobin levels on admission, median (IQR)	11 (8.9, 12.8)	11.3 (9.4, 12.9)	0.7772	9.7 (7.9, 12.6)	0.5469
Minimum hemoglobin levels, median (IQR)	9.3 (7.2, 11.3)	8.6 (7.2, 10.9)	0.6861	6.4 (5.9, 7.8)	0.0168
Blood transfusion, yes, *n* (%)	12 (32.4)	5 (33.3)	1.0000	4 (80.0)	0.0608
Units of blood transfusion, median (IQR)	0 (0, 4)	0 (0, 2)	0.9515	4 (1, 8)	0.0489
Concomitant antiplatelet agents, *n* (%)	2 (5.4)	2 (13.3)	0.5695	1 (20.0)	0.3232
Period of fasting (days), median (IQR)	3 (2, 4)	3 (2, 6)	0.8609	3 (2.5, 8)	0.4382
Period of hospitalization (days), median (IQR)	9 (7, 10)	10 (9, 16)	0.0546	16 (11, 25.5)	0.0097
Period of withdrawal of anticoagulants ≥ 5 days, *n* (%)	6 (17.7)	6 (46.2)	0.0650	2 (50.0)	0.1887
Rebleeding during hospitalization, *n* (%)	3 (8.1)	3 (20.0)	0.3382	1 (20.0)	0.4099
Readmission due to diverticular bleeding, *n* (%)	10 (27.0)	4 (26.7)	1.0000	1 (20.0)	1.0000
30-day readmission, *n* (%)	3 (8.1)	0 (0.0)	0.5480	0 (0.0)	1.0000
1-year readmission, *n* (%)	10 (28.6)	4 (26.7)	1.0000	1 (20.0)	1.0000
Early colonoscopy on admission, *n* (%)	8 (21.6)	2 (13.3)	0.7039	2 (40.0)	0.5773
Elective colonoscopy, *n* (%)	13 (35.1)	5 (33.3)	1.0000	2 (40.0)	1.0000
Interventional radiology, *n* (%)	1 (2.7)	0 (0.0)	1.0000	0 (0.0)	1.0000

*1 Appropriate standard-dose + appropriate low-dose, *2 Overdose + underdose. *Compared per-label with off-label, **compared per-label with overdose and contraindication.

Risk factors for readmission in colonic diverticular bleeding were also assessed; as shown in Table 7, our univariate logistic analysis showed that among clinical factors including age, renal dysfunction, and concomitant use of antiplatelet agents, a history of hospitalization due to colonic diverticular bleeding was the only risk factor for readmission within 1 year.

**Table 7:** Univariate logistic analysis for risk factors for readmission within 1 year.

		Odds ratio	95% CI	*P* value
Sex	Male	0.84	0.34–2.08	0.7031
	Female	Reference		
Use of DOAC	Yes	1.00	0.41–2.49	0.9920
	No	Reference		
Use of warfarin	Yes	1.00	0.40–2.47	0.9920
	No	Reference		
Concomitant use of antiplatelet agents	Yes	1.69	0.58–4.91	0.3364
	No	Reference		
Concomitant use of NSAIDs	Yes	2.37	0.88–6.34	0.0867
	No	Reference		
History of hospitalization due to LGIB	Yes	8.15	2.52–26.3	0.0005
	No	Reference		
Age (years)	≥ 80	2.07	0.81–5.29	0.1301
	< 80	Reference		
Age (years)	≥ 85	1.24	0.48–3.26	0.6565
	< 85	Reference		
CCr (mL/min)	< 60	0.85	0.30–2.39	0.7603
	> 60	Reference		
Minimum hemoglobin levels (g/dL)	< 7	3.01	1.06–8.63	0.0394
	> 7	Reference		
HAS-BLED score	≥ 3	2.83	0.95–8.44	0.0611
	< 3	Reference		

CI: confidential interval, LGIB: lower gastrointestinal bleeding caused by colonic diverticular bleeding.

## Discussion

To date, only a few studies have investigated the impact of oral anticoagulants, especially DOACs, on lower GI bleeding^14^. Brodie et al. evaluated patients with severe GI bleeding (the need for hospitalization, blood transfusion, endoscopic or surgical intervention, and 30-day mortality) treated with different oral anticoagulants and concluded that patients with severe GI bleeding who take DOACs required significantly fewer hospitalizations and fewer blood transfusions than those taking warfarin^7^. In the present study, we focused on the impact of anticoagulants on the clinical course of patients with colonic diverticular bleeding. We found the following major findings. First, the DOAC group with colonic diverticular bleeding was significantly older, with less concomitant use of antiplatelet agents in the Japanese population. The warfarin group showed significantly longer hospital stays than the DOAC group. Based on historical data, we found that the overall length of hospital stay was 11 days for patients with colonic diverticular bleeding^15^, which was similar to that of one of the DOAC groups in the present study. Second, there were no significant differences in the clinical outcomes between the per-label and off-label DOAC groups. Third, a history of hospitalization due to colonic diverticular bleeding was a significant risk factor for readmission within 1 year.

DOACs are nonvitamin K antagonist oral anticoagulants that selectively inhibit thrombin (clotting factor IIa) and factor Xa. Compared to warfarin, an antagonist of vitamin K necessary to produce multiple clotting factors (factors II, VII, IX, and X), DOACs are not affected by dietary vitamin K and are characterized by their resistance to the effects of another drug metabolism. Consequently, DOACs have recently, been used widely as the standard treatment for nonvalvular AF (NVAF). Growing evidence has supported the superiority or noninferiority of DOACs to warfarin for their antithrombotic effect and reduced bleeding risk^16^. While previous studies, including the RE-LY^4^, ROCKET-AF^6^, ARISTOTLE^5^, and ENGAGE AF-TIMI 48^17^ trials, have reported equal or lower frequencies of major bleeding or intracranial hemorrhage in AF patients receiving DOACs compared with those on dose-adjusted warfarin, they have concluded that there was statistically significant increase in GI bleeding in patients receiving DOACs. Our aim was to compare and assess the impact of two different types of oral anticoagulants, DOACs and warfarin, on lower GI bleeding. Although our study evaluated the impact of these anticoagulants on the clinical outcomes of colonic diverticular bleeding instead of measuring the risk of GI bleeding directly, we believe that the current study provided us with important insight into understanding the safety profile of DOACs. In fact, our data showed that DOAC users experienced a shorter duration of hospitalization than warfarin users. It is possible that warfarin users had longer hospitalizations compared with DOAC users because the effect of warfarin does not immediately occur after administration, and the time to stabilize the anticoagulant effect is longer for warfarin users than that for DOAC users; however, our results revealed that the duration of withdrawal of anticoagulants was also significantly longer in the warfarin group, suggesting the possibility that warfarin-associated GI bleeding was often difficult to control even after cessation of medication.

Several studies have compared the clinical impact of DOACs and warfarin in different age groups. Kirita et al. reported that there were no significant differences in the clinical characteristics of colonic diverticular bleeding (the number of recurrent bleeding events and the frequency and units of blood transfusion) between DOAC and warfarin users, even in very elderly patients who are over 80 years of age^14^. However, the present study showed that the ratio of patients over 80 years of age who required blood transfusion and the units of required blood transfusion was significantly higher in the warfarin group (Table 4). Furthermore, a comparison of the patients over and under 80 years revealed that the warfarin-treated elderly patients had significantly worse outcomes in the initial hemoglobin levels, the minimum hemoglobin levels, the ratio of patients who needed a blood transfusion, and the units of blood transfusions compared with the younger patients, suggesting that the use of warfarin in elderly patients may be harmful in terms of the management of lower GI bleeding. Interestingly, in DOAC-treated elderly patients, the rate of readmission within 1 year was significantly higher than that in younger patients. These findings suggest that there may be slight differences in the nature and characteristics between DOACs and warfarin.

There were several unique findings in the patient characteristics in the present study. Our data showed that the median age was significantly higher in the DOAC group. This finding is reasonable since PT-INR levels can be difficult to control in elderly patients because of poor adherence to medication and a reduction in the activities of daily living. In contrast, Kirita et al. reported no significant difference in the mean age between 20 warfarin and 23 DOAC patients^14^. The SAKURA AF Registry, a large-scale prospective multicenter registry designed to investigate outcomes of oral anticoagulant use in Japanese AF patients, has also reported that the mean age did not differ significantly between the two groups^18^. One of the reasons can be attributed to the fact that the registry was conducted from 2013 to 2015, which was not long since the introduction of the first commercial DOAC; therefore, physicians were not familiar with switching from warfarin to DOACs. The prevalence of concomitant antiplatelet agents differed by research; our study showed a significantly higher prevalence in patients who took warfarin, which was consistent with the results reported by Yokoyama et al*.*^19^. This result can be explained by a recent trend in favor of choosing DOACs for anticoagulant therapy and minimizing the bleeding risk in patients on antithrombotic therapy.

Our secondary endpoint focused on inappropriate prescriptions of DOACs. According to the Outcomes Registry for Better Informed Treatment of Atrial Fibrillation II (ORBIT-AF II), a significant minority (almost 1 in 8) of outpatients in the United States received DOAC doses inconsistent with the labeling^20^. In Japan, the FUSHIMI-AF Registry was the first large-scale registry study conducted from 2011 to 2015 to investigate the real-world status of anticoagulation treatment for Japanese patients with AF, and an analysis of the registry data showed that approximately 42% of the patients who took dabigatran, 27% of patients who took rivaroxaban, and 26% of the patients who took apixaban were prescribed off-label dosing of DOACs^21^. A few years later, the SAKURA AF Registry was conducted from 2013 to 2015 to investigate the real-world status and clinical outcomes of AF patients, especially those taking DOACs. According to this research, approximately 26% of the patients were prescribed off-label doses^18^. From 2011 to 2017, the DIRECT Registry, a single-center prospective observational registry of NVAF patients with DOACs, was conducted to investigate the associations between DOAC dosing and clinical features in Japanese real-world clinical practice. The registry revealed that 20% of NVAF patients received inappropriate doses of DOACs^22^. In the current study, 30% of the patients were receiving off-label doses, which was concordant with the previous results.

Off-label dosing of DOACs has attracted growing attention recently, as several studies have investigated the associations between inappropriate DOAC dosing and clinical outcomes. Arbel et al. reported that off-label dose-reduced DOAC was associated with reduced effectiveness without a safety benefit^8^. The findings from the DIRECT Registry showed that an appropriate dose reduction of DOACs was associated with a decrease in GI bleeding; however, after multivariate adjustment, there were no significant associations between DOAC dosing and GI bleeding. The current study evaluated the clinical outcomes of colonic diverticular bleeding instead of the actual risk of GI bleeding.

Several limitations should be acknowledged. First, this is a single-center retrospective study that targeted a small number of patients who were hospitalized with colonic diverticular bleeding while on anticoagulant therapy and was performed based on events that included the same recurrent patients. For this reason, limited information on the postdischarge clinical outcomes of the patients was available. Second, as is often the case with this type of study design, the clinical outcomes, such as the period of fasting and hospitalization, seemed to be affected by the clinical decisions made by the individual physicians to some extent. Therefore, we should conduct a prospective cohort study to minimize these problems and to reassess our hypotheses obtained from this study. Third, to better understand the impact of the anticoagulant drugs themselves, the effect of antiplatelet agents should also be taken into consideration. Regarding the statistical findings, it was difficult to avoid the effect of antiplatelet agents when comparing the warfarin and DOAC groups due to the small number of patients. However, the warfarin group without antiplatelet agents (*n* = 28) had significantly longer hospitalizations than the DOAC group without antiplatelet agents (*n* = 48) (12 vs. 9 days, *P* = 0.0040, data not shown), suggesting that the clinical outcomes of colonic diverticular bleeding may not necessarily be affected by concomitant use of antiplatelet agents in patients on anticoagulant therapy. We believe that the effect of concomitant antiplatelet medication on the length of hospitalization was relatively small. Similarly, Brodie et al. noted that compared with the warfarin group, GI bleeding in the DOAC group was less severe despite significantly greater concomitant aspirin use in the DOAC group compared with the warfarin group^7^. Finally, we did not examine each type of DOAC owing to the small sample size. However, unlike other DOACs, dabigatran inhibits thrombin instead of factor Xa and is an inactive prodrug that is converted to its active form in the blood and GI tract^23^. Yoshio et al. reported that dabigatran decreased the delayed bleeding rate after gastric endoscopic resection, which differed from rivaroxaban^24^. In the future, we should also focus on the type of DOAC used in patients with colonic diverticular bleeding.

In summary, our results suggest that, compared to patients taking warfarin, patients taking DOACs had a shorter hospital stay and no significant differences in the other clinical outcomes despite their advanced age. DOACs may have a more promising safety profile in managing anticoagulation in patients with colonic diverticular bleeding. The rate of inappropriate use of DOACs in the current study was comparable with previous data. It was suggested that the inappropriate use of DOACs may affect the clinical outcomes of colonic diverticular bleeding patients, and thus compliance with the standard criteria may be essential to maintain the efficacy and safety of anticoagulant therapy. Further studies are needed to evaluate the bleeding profiles of these anticoagulants in detail and to confirm our statement.

## Data Availability

The data that support the findings of this study are available upon request from the corresponding author Nishida T. The data are not publicly available due to restrictions (e.g., they contain information that could compromise the privacy of the research participants).
